# Natural Product-Derived Activity-Based and Affinity-Based Probes: Tools for Mechanism of Action Studies

**DOI:** 10.3390/molecules31071096

**Published:** 2026-03-26

**Authors:** Zhiming Shi, Hui Sun, Yi Jin, Xiaowei Xu

**Affiliations:** 1 State Key Laboratory of Natural Medicines, China Pharmaceutical University, Nanjing 211198, China; szm093612@163.com (Z.S.); sui10884@163.com (H.S.); 2 School of Engineering, China Pharmaceutical University, Nanjing 211198, China; jy071825@163.com; 3 Institute of Innovative Drug Discovery and Development, China Pharmaceutical University, Nanjing 211198, China

**Keywords:** natural product probes, label groups, ABPP, mechanism of action

## Abstract

Natural products have played a pivotal role in the history of drug discovery, yet mechanistic investigations have often posed significant challenges that impede the development of natural product-derived drugs. Traditional approaches using natural product prototypes as probes for target identification and mechanistic exploration are often hampered by labor-intensive optimization and low sensitivity, whereas the advent of advanced mass spectrometry and mature Activity-Based Protein Profiling (ABPP) strategies has propelled labeled probes derived from natural products to the forefront of research. Such probes enable efficient target identification and visualization, thereby greatly facilitating the elucidation of mechanisms of action. This article systematically introduces commonly used labeling groups, reviews recent applications of labeled probes across various classes of natural products, and aims to provide references for the study of unexplored natural products of similar types. It also outlines the development of novel photoaffinity groups, suggesting that future designs should focus on small size, strong binding affinity, and stable binding, so as to further expand their applications in chemical biology and drug discovery.

## 1. Introduction

Drug discovery based on natural products represents a historically fruitful and indispensable approach, serving as a vital source of novel therapeutic agents. Landmark discoveries such as penicillin [1,2,3], paclitaxel [4,5,6], and artemisinin [7,8] not only revolutionized the treatment of infectious diseases, cancers, and malaria but also continue to guide drug development as invaluable lead compounds. Despite a shift in the pharmaceutical industry toward high-throughput synthetic libraries since the 1990s [9], natural products remain irreplaceable for exploring unique chemical scaffolds and bioactivities, with ongoing research dedicated to uncovering new therapeutic breakthroughs from nature.

**Scheme 1:**
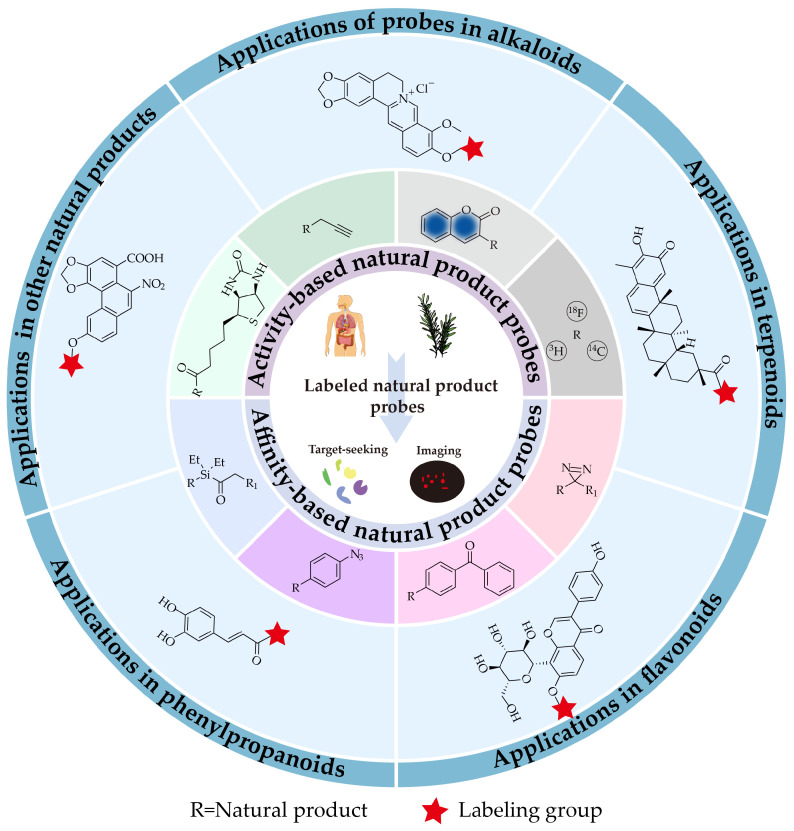
Natural products derived from plants or animals can be modified into labeled probes for use in mechanistic studies, such as target identification and imaging. Labeled natural products can be divided into two types: activity-based probes and affinity-based probes. Commonly used labels include biotin, alkyne groups, fluorophore and isotope, and photoaffinity labels like diazirine, benzophenone, and aryl azide, as well as novel photoaffinity groups such as acyl silanes. These labeling strategies have been widely applied in the study of alkaloids, terpenoids, flavonoids, phenylpropanoids, and other classes of natural products.

**Figure 1:**
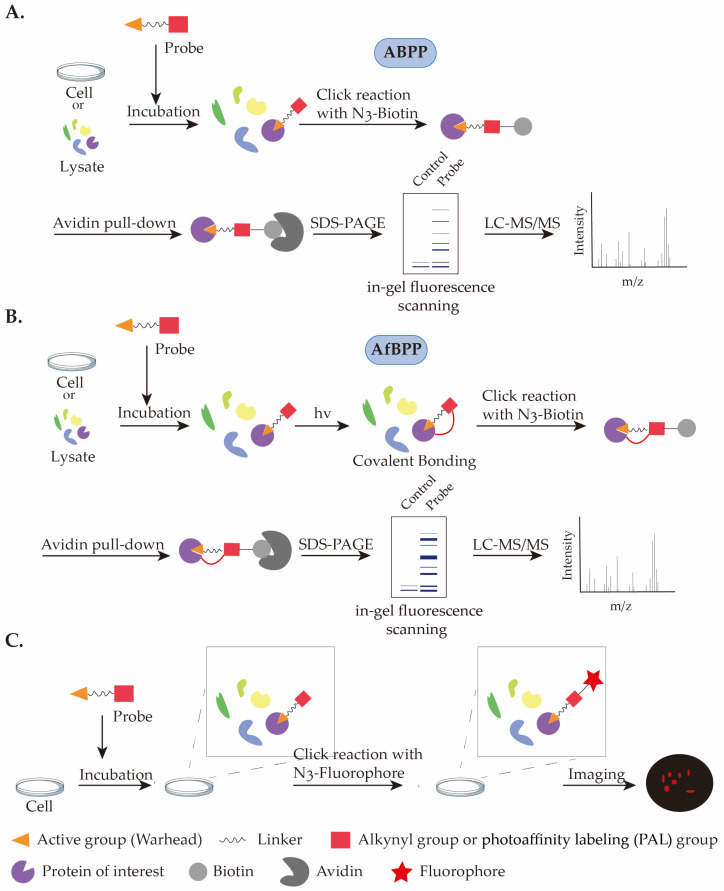
(**A**) Workflow for target identification using ABPP. (**B**) Workflow for target identification using AfBPP. (**C**) Workflow for imaging using natural product-based probes.

Elucidating the molecular targets and mechanisms of action (MOA) of bioactive natural products is a critical step in advancing their development into therapeutics (Scheme 1). In many studies, natural products can be directly employed as probes for target identification. For example, recent research demonstrated that Walrobsin A (WA), a compound derived from Walsura robusta, significantly alleviates septic acute kidney injury (S-AKI) in mice. Using WA as a probe in combination with Drug Affinity Responsive Target Stability–Mass Spectrometry (DARTS-MS), the authors identified GPR75 as its direct target and further clarified the underlying mechanism through subsequent experiments [10]. Such label-free approaches preserve the native activity of the natural product without interfering with protein binding; however, they often require extensive optimization and exhibit relatively low sensitivity in protein identification [11]. Activity-based protein profiling (ABPP) and affinity-based protein profiling (AfBPP) are widely used techniques for target discovery [12,13,14]. These strategies involve site-specific labeling of natural products to enable enrichment and identification of bound target proteins. With established experimental workflows and enhanced detection sensitivity, ABPP and AfBPP have become efficient tools for target deconvolution. Taking the application of an alkyne-containing orthogonal probe in ABPP as an example: first, the probe is incubated with live cells or cell lysates to allow sufficient interaction with target proteins; then, azide-biotin is introduced into the system, enabling biotinylation of the probe via click chemistry; subsequently, streptavidin is used to enrich the biotin-labeled probe–protein complexes; the enriched protein mixture is separated by sodium dodecyl sulfate–polyacrylamide gel electrophoresis (SDS-PAGE), and target proteins are finally identified and analyzed by LC-MS/MS (Figure 1A,B). Such probes can also be adapted for fluorescent labeling in live-cell cultures, allowing spatial localization and imaging of the probe’s site of action (Figure 1C) [15].

Rational design of labeled probes derived from natural products is essential for the successful and accurate identification of targets and elucidation of their mechanisms of action using proteomics-based approaches. Natural products often possess diverse pharmacophores [16] and contain multiple modifiable functional groups (such as hydroxyl and carboxyl groups). However, in the absence of clear structure-activity relationships, the impact of modifying different sites on biological activity remains uncertain. Therefore, in the early stages of research, it is common practice to synthesize multiple probe molecules targeting various potential modification sites. These probes are then screened and validated through phenotypic assays [17,18] and competitive binding experiments with the native natural product to identify those that retain activity. Beyond the choice of modification sites, the properties of the tagging group itself such as size, hydrophilicity/hydrophobicity, and reactivity can significantly influence the biological activity and pharmacokinetic profile of the probe [19,20]. Thus, the selection of an appropriate tagging group is also a critical aspect of probe design. This review first provides an overview of commonly used tagging groups, including biotin, alkyne, fluorophore, isotope, diazirine, benzophenone, and aryl azide, and highlights recent advances in novel photoaffinity groups such as acyl silanes. It then summarizes recent applications (2021–2025) of labeled probes in target discovery across different classes of natural products, including alkaloids, terpenoids, flavonoids, phenylpropanoids and other natural products. The aim is to offer methodological insights and design considerations for future mechanistic studies on unexplored natural products of similar structural classes.

## 2. Activity-Based Natural Product Probes

Activity-based probes (ABPs) serve as key tools in chemical biology for elucidating the mechanisms of action of natural products. Such probes typically consist of three core functional modules: first, the active group (warhead), derived from the parent natural product structure and retaining its ability to specifically bind to the active site of the target protein; second, the reporting group or label, used for visual detection or affinity-based enrichment of the target; and finally, a linker that covalently connects the two. The length and chemical properties of the linker must be rationally designed to minimize steric hindrance, thereby preserving the probe’s biological activity and permeability. In practice, the choice of reporting group, such as fluorophores, biotin, or click chemistry handles, requires careful consideration of the specific research objectives and its potential impact on the inherent pharmacological activity of the natural product.

### 2.1. Biotin Group

**Figure 2:**
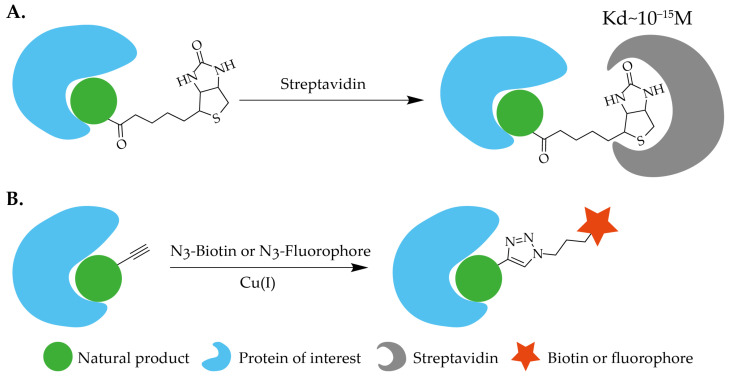
(**A**) Biotin-labeled natural product probes combined with streptavidin for protein enrichment. (**B**) Alkyne-labeled natural product probes conjugated with biotin-azide or fluorophore-azide via click chemistry to form triazole linkages for protein enrichment or imaging.

Biotin (also known as vitamin B7) is a key cofactor in cellular metabolism, involved in the synthesis of amino acids and carbohydrates. In target discovery research of natural products, covalently linking biotin to natural product molecules is a commonly used probe design strategy. The core of this strategy lies in utilizing the ultra-high affinity and specific binding between biotin and streptavidin, with a binding constant as high as 10^−15^ M, which is one of the strongest non-covalent interactions known to date [21]. Based on this property, biotin probes are widely used for the isolation and enrichment of target proteins: they can either be pre-immobilized on streptavidin affinity resin to adsorb and purify target proteins from cell lysates; or the probes can first be incubated with live cells or lysates to label the target proteins, followed by capture of the formed probe-protein complexes using streptavidin-based media (Figure 2A). When designing such probes, the following points require special attention: First, due to the inherent steric bulk of the biotin molecule, it is usually necessary to introduce a linker of appropriate length and flexibility between biotin and the active moiety of the natural product to minimize steric hindrance and preserve the original biological activity of the natural product as much as possible. Second, biotin itself has poor water solubility, and its derivatization often significantly reduces the solubility of the entire probe molecule. Therefore, the solubility conditions of the probe must be carefully considered and optimized in the experimental design. Furthermore, given that biotin itself participates in cellular metabolic processes, to rule out potential nonspecific background or interference, it is crucial to include negative control groups containing only biotin or the corresponding linker in the experiments.

### 2.2. Alkynyl Group

Alkyne-modified natural product probes represent a class of functional molecules constructed based on the principles of click chemistry. These probes typically employ a two-step strategy: first, a probe precursor containing only an alkyne without a reporter unit is incubated with cell lysates or living cells to enable specific binding to target proteins; subsequently, a reporter group (biotin) is introduced via a highly efficient and selective click chemistry reaction, thereby facilitating the enrichment and detection of the target protein (Figure 2B). Click chemistry allows for the efficient formation of heteroatom linkages under mild conditions and has been widely adopted in medicinal chemistry and chemical biology since its conceptual inception [22]. Currently, the most commonly utilized bioorthogonal click reactions are predominantly cycloadditions, which proceed without cleavage of σ-bonds; these mainly include copper-catalyzed azide-alkyne cycloaddition (CuAAC), strain-promoted azide-alkyne cycloaddition (SPAAC), and inverse electron-demand Diels-Alder reactions (IEDDA). From a synthetic perspective, alkyne modification of natural products is generally more straightforward and safer than azide modification and is compatible with a wider range of commercially available reporter tags, making it suitable for versatile applications in target discovery scenarios such as cellular imaging and proteomics. The alkyne group offers advantages including small molecular volume and high biological inertness, minimizing interference with the inherent activity and in vivo behavior of the natural product. Owing to these combined strengths, alkyne-modified probes are widely used in research, with the technically mature and mild CuAAC reaction being particularly frequently reported in the relevant literature.

### 2.3. Fluorophore

**Figure 3:**
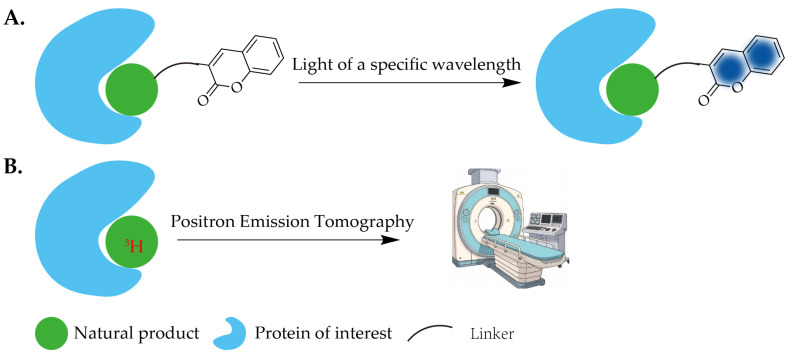
(**A**) Upon excitation by specific wavelengths of light, natural product probes containing the coumarin fluorophore emit fluorescence for imaging purposes. (**B**) Radiolabeled natural product probes facilitate both imaging and target identification via PET technology.

Except for a limited number of natural products that possess intrinsic luminescent properties, the introduction of fluorophores has become a standard strategy for enabling the bioimaging of non-luminescent natural products. Two primary methods are commonly employed for fluorophore incorporation. The first involves direct conjugation of a fluorophore to the natural product, either with or without a linker, to generate a fluorescent probe (Figure 3A). The second approach entails the initial synthesis of an orthogonal probe derived from the natural product. Following specific binding to the target within cells or tissues, the fluorophore is introduced via click chemistry, facilitating in situ imaging. Commonly used fluorophores include Cy3, coumarin, and FITC, etc. [23]. Upon excitation with light of a specific wavelength, these fluorophores absorb photon energy, leading to an electronic transition to an excited state. Subsequent relaxation back to the ground state results in the emission of light at a longer wavelength, a phenomenon known as fluorescence. However, due to factors such as the relatively large molecular size, potential intrinsic bioactivity, and solubility characteristics of these fluorophores, probes constructed via the first method often exhibit compromised cell permeability and may suffer from interference with the native biological activity of the natural product [24]. In contrast, the second method is less susceptible to these limitations and is therefore more widely adopted for biological applications.

### 2.4. Isotope Labeling

The introduction of isotopic labels into natural products constitutes an important strategy for investigating their mechanisms of action. The direct incorporation of radioactive isotopes, such as ^3^H or ^14^C, into natural product scaffolds is commonly employed for target discovery and in vivo tracing studies (Figure 3B). Furthermore, ^18^F-labeled natural product derivatives are widely utilized in diagnostic imaging for diseases [25]. However, this approach has practical limitations: site-specific incorporation often poses significant synthetic challenges, and the half-lives of the radionuclides typically result in narrow experimental windows. An alternative strategy involves the metabolic incorporation of stable isotopes, such as ^2^H or ^13^C-labeled amino acids, during cell or tissue culture (e.g., via SILAC or iTRAQ/TMT techniques). This leverages the organism’s native protein synthesis machinery to achieve metabolic labeling of the proteome. Subsequently, target proteins are enriched using Activity-Based Protein Profiling (ABPP) or Affinity-Based Protein Profiling (AfBPP). Coupled with mass spectrometry for relative quantification of protein abundance, this enables quantitative proteomics analysis. This strategy, owing to its high throughput, unbiased nature, high resolution, and quantitative accuracy, has been widely adopted in the field of natural product target discovery.

## 3. Affinity-Based Natural Product Probes

Although a few natural products are capable of covalent target binding, most interactions are non-covalent and often transient. Consequently, if the binding affinity is too low to persist through proteomic sample preparation, the activity-based probe (ABP) strategy may lead to the omission of key proteins or even fail to achieve target identification [11]. The development of photoaffinity labeling (PAL) and the application of AfBPP have addressed this limitation of activity-based protein profiling ABPP by introducing photoreactive groups into probes, enabling covalent binding between the probe and target proteins. Given the needs of target identification and imaging, affinity-based probes (AfBPs) often carry an alkyne handle for click chemistry reactions. In a study aimed at identifying the targets of microbial-derived indole metabolites [26], Zhao and his team designed and synthesized the click chemistry probe alk-IAA and the photoaffinity probe x-alk-IAA for indole-3-acetic acid. Both probes were incubated with HT-29 cells, followed by UV irradiation and enrichment of the bound proteins. In-gel fluorescence analysis showed that alk-IAA bound only to a limited number of proteins, whereas the x-alk-IAA group exhibited extensive protein labeling in the range of 20 kDa to 75 kDa. This result demonstrates the superior protein-binding capability of the photoaffinity probe. Currently, commonly used photoaffinity probes mainly include diazirine probes, benzophenone probes, and aryl azide probes. The selection of an appropriate probe primarily depends on factors such as the size of the photoaffinity group, the wavelength and duration of irradiation, and the lifetime of the reactive intermediate.

### 3.1. Diazirine Group

Diazirine is a commonly used photoreactive group. Upon irradiation with specific wavelengths of ultraviolet light, it releases a molecule of nitrogen to generate a highly reactive carbene intermediate. This carbene can form covalent bonds with amino acids in proteins, particularly with the side chains of polar amino acids (Figure 3) [27]. Due to the high reactivity of the intermediate, non-specific conjugation between the probe and proteins often occurs almost instantaneously, while side reactions such as self-reaction of the probe may also take place. In practical applications, diazirines are categorized into aliphatic and aromatic types. Aliphatic diazirines are more widely used due to their smaller steric bulk. To minimize interference with protein binding, Yao’s team proposed the concept of “minimalist probes.” They developed a linear, terminal alkyne-containing diazirine-based photo-crosslinker [28], which has gained extensive application.

### 3.2. Benzophenone Group

**Figure 4:**
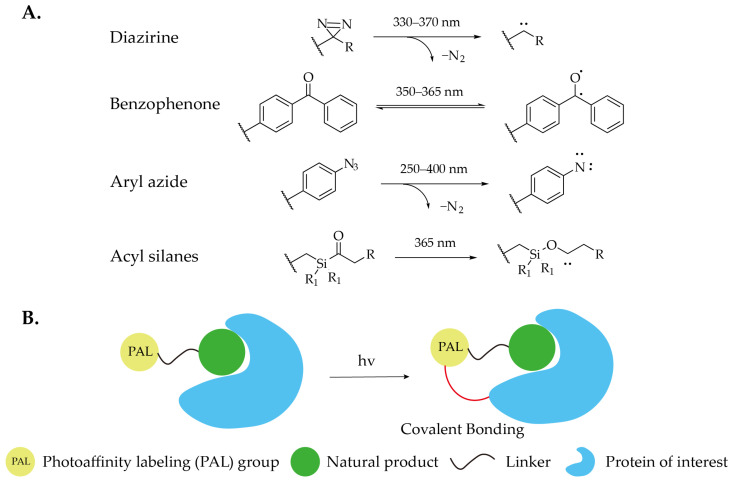
(**A**) Photoaffinity groups such as diazirine, benzophenone, aryl azide, and acyl silanes are activated upon exposure to UV light at specific wavelengths. (**B**) PAL enables covalent binding with proteins upon light activation.

Benzophenone is a classic photochemical crosslinking group. Upon irradiation with long-wavelength ultraviolet light (typically ~365 nm), its carbonyl group can be activated into a reactive diradical species, which then forms covalent crosslinks with proteins (Figure 4). In photoaffinity labeling of proteins, this group exhibits good selectivity and affinity for methionine side chains, making it valuable for mapping protein interaction interfaces [29]. However, benzophenone has notable limitations in practical applications. Compared to other photo-crosslinking groups such as diazirines, benzophenone generally requires longer irradiation times for photoactivation. More importantly, due to its relatively large molecular volume and rigid structure, when directly attached to small-molecule natural products, the bulky photoactive moiety can severely interfere with the normal binding between the bioactive molecule and its target protein, potentially leading to loss of biological activity. This limits its broad application in photoaffinity probes derived from natural products. To overcome these issues of steric hindrance and interference with activity, a common strategy is to introduce a longer linker between the benzophenone unit and the active molecule. By rationally designing the length and chemical properties of the linker, it is possible to reduce the spatial and electronic interference caused by the photo-crosslinking group on the active moiety, thereby better preserving the original biological activity of the probe. The design and optimization of linkers have thus become a key aspect in improving the performance of such photoaffinity probes.

### 3.3. Aryl Azide Group

Aryl azides represent a class of photoaffinity reagents that, upon ultraviolet irradiation, also release a nitrogen molecule to generate a highly reactive nitrene intermediate, leading to nonspecific covalent binding with proteins (Figure 4). Compared to the two aforementioned types of photoaffinity groups, aryl azides generally require shorter excitation wavelengths and exhibit relatively lower photoreaction efficiency. Consequently, their application scope is not as broad as that of diazirine- and benzophenone-based probes, and they are primarily employed for specific modifications of endogenous aromatic compounds such as phenylalanine. In a recent study aimed at identifying cellular targets of lysine demethylases (KDMs), a photoactive azido-methylamino system was engineered deep within the active site of KDM [30]. The azido-modified phenylalanine derivative p-azido phenylalanine (AzF) was incorporated into the enzyme. The resulting photoactive mutant KDM4A-171AzF was incubated with lysates of HEK293T cells treated with n-octyl-IOX1, followed by irradiation at 365 nm to covalently cross-link the probe with interacting proteins. After elution and isolation of the bound proteins, further proteomic analysis revealed that a range of non-histone proteins also serve as substrates for KDM.

### 3.4. Novel Photoaffinity Moieties

Classic photoaffinity probes are widely used, and the development of novel photoaffinity probes has never ceased. Page and his team are dedicated to developing small molecules that target “undruggable” proteins using chemoproteomic approaches. The team successfully constructed an acyl silane probe [31], which features an acyl silane structure and a terminal alkyne group. Upon UV irradiation, the photoaffinity activity of the probe is activated, and the acyl silane moiety undergoes a photo-Brook rearrangement to generate an α-siloxy carbene (Figure 4), which covalently binds to target proteins, enabling their identification through subsequent proteomic analysis. To validate the functionality of the acyl silane probe, the researchers generated acyl silane probes JQ1-iPr and JQ1-Et based on the BET inhibitor JQ1, and synthesized a diazirine probe JQ1-DA as a control. The study demonstrated that the acyl silane probe bound to more proteins and exhibited higher selectivity compared to the diazirine probe [32]. Nonetheless, acyl silane probes have demonstrated excellent protein-binding capabilities. With continued optimization, their potential in target discovery is poised to be fully realized.

## 4. Application of Probes in Natural Product Research

### 4.1. Research on Alkaloids

Alkaloids are naturally occurring, low-molecular-weight nitrogenous compounds that are primarily biosynthesized from amino acids in organisms [33]. Based on their structural skeletons, alkaloids can be categorized into isoquinoline, indole, pyridine, and other types. These natural products exhibit broad biological activities and show promising potential in areas such as anticancer [34,35], antibacterial [36], and neuroprotective effects [37] (Table 1).

Berberine (BBR), an isoquinoline alkaloid derived from *Coptis chinensis*, exhibits a wide range of pharmacological activities. To investigate its anti-inflammatory mechanism, Wei’s team employed benzophenone as the photoaffinity group to design and synthesize an alkyne-containing BBR affinity probe (probe 1) [38]. Compared to BBR, probe 1 exhibited superior inhibitory activity against interleukin-1β (IL-1β). Following co-incubation with PMA-induced THP-1 cells and activation under 365 nm UV light, the probe-protein conjugates were conjugated with azide-biotin via click chemistry, enriched by streptavidin, and separated by SDS-PAGE. Proteomic analysis based on probe 1 identified 44 potential targets. Among these, the Gene Ontology (GO) enrichment analysis indicated that the function of eukaryotic translation initiation factor 2 alpha kinase 2 (EIF2AK2) is highly correlated with the known indications of BBR, and both cellular thermal shift assay (CETSA) and molecular docking indicated the strongest affinity between EIF2AK2 and BBR. Further studies demonstrated that BBR exerts its anti-inflammatory effects by inhibiting the dimerization of EIF2AK2 and selectively regulating downstream signaling pathways. In another study by Zeng’s team, they synthesized photoaffinity probes containing aryl diazirine based on BBR [39]. Probe 2, with longer linkers, exhibited better performance within the series and revealed that BBR exerts its activity by targeting MAP2K7 and inhibiting phosphorylated JNK.

**Figure 5:**
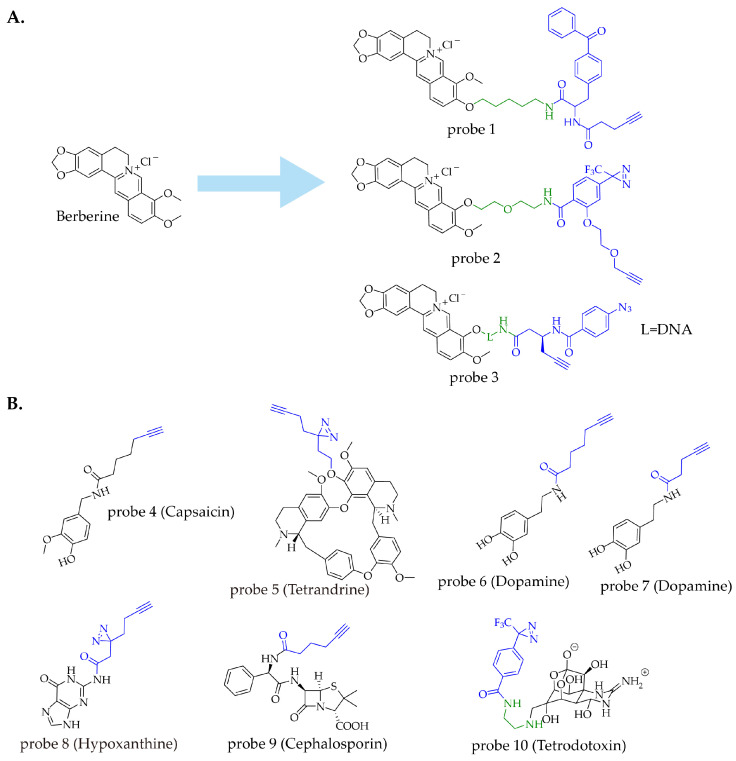
(**A**) Three berberine-based photoaffinity probes modified with benzophenone, aryldiazirine, and aryl azide, respectively. (**B**) The structures of labeled probes derived from other alkaloid-based natural products.

To investigate the hypoglycemic mechanism of BBR, Qi’s team employed a DNA-programmed affinity labeling (DPAL) strategy to construct a dual-probe system (probe 3). This system consisted of a binding probe (BP), in which berberine was directly conjugated to an oligonucleotide strand, and a capture probe (CP), where a photoaffinity group was linked to a complementary oligonucleotide strand. In the experiment, cells were first incubated with the BP, followed by the addition of the CP, allowing the two probes to efficiently associate near the target via complementary DNA hybridization. The study demonstrated that BBR exerts its hypoglycemic effect by targeting the TIGAR protein and inhibiting the conversion of fructose-2,6-bisphosphate to fructose-6-phosphate [40]. This strategy, which uses DNA as a programmable linker, effectively mitigates issues such as poor labeling efficiency and high background interference commonly associated with conventional probes, offering a novel approach for probe design. For different indications of BBR, all three probes (Figure 5A) were modified and connected at similar phenolic ether positions, indicating that the active structure of BBR exhibits similarity in its interaction with the target proteins across these three studies. The selection of different optimal tagging groups in each case is primarily attributed to variations in the microenvironment surrounding the active site of the target proteins, such as spatial constraints and the types of amino acids present.

Capsaicin (CAP), a natural product derived from *Capsicum annuum* L., exhibits a variety of biological activities, including analgesic [49], anticancer [50], and antibacterial [51] effects. Zhang’s team designed and synthesized a series of orthogonal probes based on CAP. Among them, probe 4, in which the side chain of CAP was replaced with an alkyne group, retained CAP’s bioactivity and was employed for target identification (Figure 5B) [41]. The team identified PKM2, LDHA, and COX-2 as direct targets of CAP in RAW264.7 cells, revealing that CAP alleviates the Warburg effect and inflammation in sepsis by simultaneously targeting PKM2-LDHA and COX-2. In another mechanistic study by He’s group on CAP’s effect against rheumatoid arthritis (RA) [42], the same orthogonal probe (probe 4) was used and led to the discovery of PRDX2 as a target. The research demonstrated that CAP binds to PRDX2, inhibits its antioxidant capacity, and subsequently activates oxidative stress and apoptosis pathways, thereby exerting anti-arthritic effects. These two studies suggest that modification of CAP’s side chain has little impact on its anti-inflammatory activity, while also indicating the feasibility of applying the same probe across different disease models for mechanistic investigation.

Tetrandrine (Tet), a bisbenzylisoquinoline alkaloid extracted from the plant *Stephania tetrandra* S. Moore [52], is a potent inhibitor of Ebola virus replication. Its mechanism involves blocking NAADP-dependent calcium release mediated by lysosomal two-pore channels (TPCs) [53]. To investigate the molecular targets and mechanism of Tet’s antiviral action, Chan’s team designed and synthesized a minimally modified, diazirine-labeled Tet probe (probe 5) (Figure 5B) [43]. The authors first labeled probe 5 with a BODIPY fluorophore and confirmed through competitive binding assays and fluorescence analysis that the probe retained Tet’s biological activity. Confocal imaging revealed that probe 5 primarily accumulated in cellular lysosomes. Subsequently, the study employed stable isotope labeling by amino acids in cell culture (SILAC) [54] combined with quantitative proteomic analysis, identifying over 700 proteins. Among these, 12 proteins showing significantly high signals in mass spectrometry were identified as potential targets of probe 5, all of which were localized to lysosomes. Using siRNA knockdown screening, the authors further confirmed the lysosomal membrane protein LIMP-2 as a key target of Tet and elucidated that Tet is recruited to the lysosomal membrane via a mechanism dependent on LIMP-2. This study fully leveraged the imaging and target-capture capabilities of probe 5. The quantitative proteomics strategy visually presented the binding between proteins and the probe in a data-driven manner, which not only shortened the target identification timeline but also significantly enhanced the credibility of potential target discovery.

To investigate the mechanisms underlying the anticancer and neuroprotective effects of catechol-containing natural products in vivo, Muñoz’s team synthesized a dopamine probe with broad reactivity toward catechol groups [44]. The researchers linked an alkyne handle to the amino group of dopamine via an amide bond, a design that avoids side reactions such as cyclization or polymerization involving the amino group while preserving the reactivity of the catechol moiety. Three probes with different linker lengths were synthesized. In-gel fluorescence analysis showed that probe 6, with a seven-carbon chain, exhibited the strongest labeling signal in a concentration-dependent manner. Follow-up studies revealed that endoplasmic reticulum (ER) proteins are major targets of catechol-type compounds. Analysis of ER stress in the presence of reactive catechols showed activation of the unfolded protein response (UPR), providing a mechanistic explanation for the bioactivity of catechol-containing natural products. Chen’s team designed and synthesized a dopamine probe with a shorter side chain, probe 7 [45]. Using this probe, experiments identified three glycolytic enzymes—aldolase A, α-enolase, and pyruvate kinase M2—as direct targets of dopamine. This finding suggests that dopamine acts on these proteins to block ATP production, thereby leading to mitochondrial dysfunction. Hypoxanthine (Hx) is a purine metabolite and a precursor of uric acid. Huang’s team designed and synthesized the photoaffinity probe (probe 8) for Hx [46], and its activity was validated through competitive experiments with Hx in insulin-resistant HepG2 (IR-HepG2) cells. Using proteomic analysis followed by bioinformatics screening, 43 highly credible protein targets were identified. KEGG enrichment analysis showed that these proteins are involved in various metabolic pathways in the liver. Experiments demonstrated that Hx regulates glucose and lipid metabolism via the AMPK/mTOR/PPARα pathway, thereby alleviating insulin resistance in IR mice.

For the development of probes targeting these two animal-derived endogenous alkaloids mentioned above, the design philosophy is consistent with that for plant-derived alkaloids. Further examples include cephalosporin (probe 9) [47] and tetrodotoxin (probe 10) [48]. The fundamental principle of this strategy can be summarized as: minimize structural modification while maximizing the retention of activity (Figure 5B).

### 4.2. Research on Terpenoids

Terpenoids represent the most abundant class of secondary metabolites in nature, with isoprene serving as their fundamental structural unit. The biosynthesis of terpenoids primarily occurs via two pathways: the mevalonic acid (MVA) pathway and the methylerythritol phosphate (MEP) pathway [55]. Most terpenoid compounds have been demonstrated to possess bioactivities such as anti-inflammatory, anticancer, and antimicrobial effects [56,57,58] (Table 2).

Ganoderic acid T (GAT), a triterpenoid compound isolated from *Ganoderma lucidum*, has been reported to exhibit various antitumor activities [71]. To investigate its antitumor mechanism, Lei et al. coupled GAT with biotin via an ethylenediamine linker to construct the probe Biotin-GAT (probe 11). Experiments showed that probe 11 retained antiproliferative activity similar to that of GAT. After incubating probe 11 and a negative control (free biotin) separately with Hep3B cell lysates, streptavidin-agarose beads were used to capture the bound proteins, which were then separated by SDS-PAGE and visualized by silver staining. The results revealed that probe 11 specifically pulled down a protein band of approximately 130 kDa, which was subsequently identified by mass spectrometry as pyruvate carboxylase (PC), suggesting PC as a potential target of GAT. Further studies demonstrated that GAT specifically targets PC, disrupts glycolysis and the tricarboxylic acid cycle in hepatocellular carcinoma cells, and induces apoptosis through the ROS-mediated JNK/p38 MAPK signaling pathway [59]. In another study, pedunculoside (PE), a triterpenoid saponin extracted from *Ilex rotunda* Thunb., was found to exhibit activity in preventing pathological cardiac hypertrophy, although its mechanism remained unclear. Pan et al. employed succinic diamine as a linker to conjugate PE with biotin via amide bonds, synthesizing the probe biotin-PE (probe 12). After incubating the probe with neonatal rat cardiomyocyte (NRCM) lysates, a pull-down assay was performed, leading to the identification of the transcription factor GATA6 as its target. Subsequent research confirmed that PE exerts its protective effects by targeting GATA6 [60]. Both studies utilized biotin as the reporter group and shared considerable similarity in probe design strategy, indicating that biotin-based labeling holds good applicability and potential for target identification in triterpenoid natural products.

Celastrol, a natural product extracted from *Tripterygium wilfordii*, exhibits diverse biological activities. Researchers synthesized the probe Cel-P (probe 13) by linking celastrol to propargylamine via an amide bond [61]. Proteomic analysis and cellular thermal shift assays based on this probe demonstrated that celastrol binds to both peroxiredoxins (PRDXs) and heme oxygenase 1 (HO-1). Binding to PRDXs inhibits their antioxidant activity, while binding to HO-1 upregulates its expression, jointly promoting the accumulation of reactive oxygen species (ROS) and inducing ferroptosis in activated hepatic stellate cells (HSCs), thereby exerting anti-fibrotic effects in the liver. In another study by Shi’s team [62], probe 13 was used to identify mitochondrial isocitrate dehydrogenases (IDHs) as targets. The research showed that celastrol binds to and inhibits IDH activity, disrupts mitochondrial metabolism, subsequently suppresses the DPYSL2-JAK/STAT signaling pathway, and ultimately promotes apoptosis and ferroptosis in breast cancer cells. Furthermore, the same probe (probe 13) identified PfSpdsyn and PfEGF1-α as key targets through which celastrol exerts its anti-malarial effects in Plasmodium falciparum [63]. In a separate study conducted by Shang’s team [64], the same probe (probe 13) was employed to identify the target protein AMOTL2. It was revealed that celastrol binds to AMOTL2, promotes the degradation of YAP1, inhibits PGC-1α/TFAM-mediated mitochondrial biogenesis, and ultimately leads to cardiomyocyte apoptosis, manifesting cardiotoxicity. Although celastrol is also a pentacyclic triterpenoid, it does not require a long carbon-chain linker to maintain its activity in probe design. This suggests that its binding to target proteins may be relatively shallow, so that only a partial structural fragment is sufficient for effective binding, or that the binding pocket is located on the protein surface with a relatively flat surrounding environment. This characteristic also provides potential insights for lead optimization and drug development based on partial active structural motifs of celastrol.

**Figure 6:**
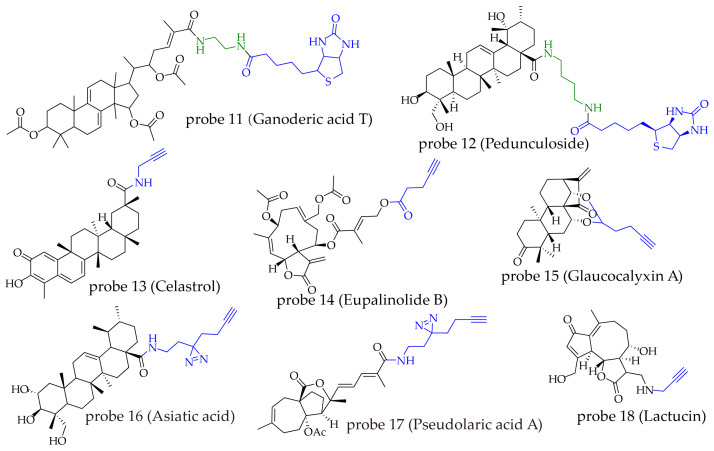
The structures of labeled probes derived from terpenoid-based natural products.

Eupalinolide B (EB) is a sesquiterpene lactone compound derived from *Eupatorium lindleyanum*, exhibiting significant anti-inflammatory and anticancer activities [72,73]. The research team constructed probe 14 by linking EB via an ester bond to a carbon chain bearing a terminal alkyne group [65]. Proteomic analysis based on this probe identified branched-chain amino acid transaminase 1 (BCAT1) as the direct target of EB in triple-negative breast cancer (TNBC) cells. By targeting BCAT1, EB reduces the synthesis of branched-chain amino acids, thereby inhibiting SHOC2 expression and the RAS-ERK signaling pathway, ultimately inducing apoptosis in TNBC cells. In another study focusing on periodontal inflammation [66], the same probe (probe 14) was used to identify UBE2D3 as the direct target of EB in lipopolysaccharide-activated RAW264.7 cells. Through targeting UBE2D3, EB inhibits the ubiquitination and degradation of IκBα, thereby suppressing the activation of the NF-κB signaling pathway and exerting anti-inflammatory effects. Glaucocalyxin A (GLA) is a diterpenoid compound isolated from *Rabdosia japonica*. Using an orthogonal probe strategy (probe 15), it was found that GLA covalently targets the peroxiredoxin proteins PRDX1/2, thereby modulating downstream autophagy pathways and inhibiting the progression of renal cell carcinoma [67]. Asiatic acid (AA) is a pentacyclic triterpenoid compound obtained from *Centella asiatica*. By employing a minimalist probe containing a diazirine group (probe 16), research revealed that AA exerts anti-hepatocellular carcinoma effects by targeting α-tubulin TUBA1B [68]. Similarly, using a minimalist probe strategy (probe 17), the diterpenoid pseudolaric acid A (PAA), extracted from the bark of Pseudo-larix, was found to bind to MTHFD1L, inducing the accumulation of reactive oxygen species (ROS) and thereby exerting antitumor effects [69]. Imam’s team identified four key target proteins—PGM, PKM, LDHA, and PDH—using the Lactucin probe (probe 18) combined with proteomic analysis, and revealed that Lactucin inhibits the proliferation of lung cancer cells by targeting these proteins to modulate central carbon metabolism [70]. In these studies, orthogonal probes and minimalist probe strategies have been widely applied. The results indicate that tagging groups with small molecular volumes have minimal impact on compound activity, offering greater advantages in probe design (Figure 6).

### 4.3. Research on Flavonoids

Flavonoids are a class of secondary metabolites widely distributed in plants. Their basic structure consists of a C6-C3-C6 three-ring framework formed by two benzene rings (rings A and B) connected via a pyran ring (ring C). In plants, ring A and ring B are generally synthesized through the acetate-malonate pathway and the shikimate pathway, respectively, and are subsequently cyclized by chalcone isomerase to form the characteristic flavonoid skeleton [74]. This class of compounds exhibits structural diversity and demonstrates a broad range of biological activities [75] (Table 3).

Puerarin can significantly reduce fat absorption, yet its specific molecular target remains unclear. To elucidate its mechanism, researchers synthesized a photoaffinity probe (probe 19) [76]. This probe was constructed by attaching a linear tag containing both a diazirine photo-crosslinking group and a terminal alkyne to the puerarin structure. Experiments confirmed that the probe retained puerarin’s ability to increase fecal lipid excretion. In the study, the probe was incubated with brainstem lysate and subjected to 405 nm ultraviolet irradiation for 10 min to activate the photo-crosslinking reaction, enabling covalent binding between the probe and its target proteins. Subsequently, a copper-catalyzed azide-alkyne cycloaddition (CuAAC) reaction was used to conjugate azide-biotin to the probe-protein complexes. The complexes were then purified using an avidin column, and the enriched binding proteins were identified by LC-MS and verified by Western blotting. Among the 571 potential target proteins captured, only 14 belonged to membrane receptors or ion channels, with the γ-aminobutyric acid type A receptor α1 subunit (GABRA1) identified as the most promising candidate. Further mechanistic studies revealed that puerarin exerts its inhibitory effect on the dorsal motor nucleus of the DMV–vagus axis by binding to GABA_A_ receptors containing the α1 subunit, thereby regulating fat absorption. Notably, the same probe also yielded significant findings in another study on cardiovascular disease [77]. Puerarin exhibits protective effects on cardiomyocytes. To investigate the underlying mechanism, Huang’s team synthesized probe 19 and confirmed that it possesses cardioprotective activity similar to that of puerarin. Using AfBPP for target pull-down and identification, the study found that puerarin binds to chromatin assembly factor 1B (CHAF1B) and exerts its cardioprotective effect by inhibiting cardiomyocyte apoptosis.

Norathyriol (NT) is a tetrahydroxyflavone compound isolated from *Mangifera indica* L., exhibiting various biological activities, including anti-inflammatory, antitumor, and antioxidant effects [80]. Wang’s team discovered that NT inhibits miRNA activity and designed and synthesized an orthogonal probe (probe 20) for NT [78]. Experiments demonstrated that AGO2 is the direct target of NT; NT suppresses miRNA function by preventing the loading of a set of distinct miRNAs onto AGO2. In another study conducted by Dong’s team (probe 21), it was found that bavachinin (BVC) promotes liver regeneration and alleviates non-alcoholic fatty liver disease by targeting proliferating cell nuclear antigen (PCNA) and enhancing its interaction with DNA polymerase δ [79].

**Figure 7:**
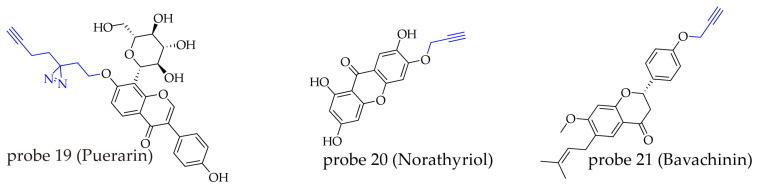
The structures of labeled probes derived from flavonoid-based natural products.

In the studies mentioned above, tagging groups were introduced via a straightforward nucleophilic substitution reaction, specifically the Williamson ether synthesis, with the phenolic hydroxyl groups of flavonoid natural products. The substitution of phenolic hydroxyl groups did not lead to significant loss of activity, suggesting that the bioactivity of flavonoids may primarily originate from their core scaffold, while the phenolic hydroxyl groups contribute relatively little to the activity. Moreover, the high reactivity of phenolic hydroxyl groups also facilitates the construction of probes in a simpler and more efficient manner (Figure 7).

### 4.4. Research on Phenylpropanoids

Phenylpropanoids are a widely distributed class of compounds in plants, primarily derived from aromatic amino acids and characterized by a C6-C3 structure containing a benzene ring. They serve as important metabolic intermediates and can be further converted via the phenylpropanoid pathway into various metabolites such as flavonoids and lignans [81]. These compounds also exhibit diverse and significant biological activities [82] (Table 4).

Caffeic acid (CA) is a phenolic acid compound widely present in tea leaves and coffee beans [90]. Zhu’s team found that CA can inhibit the production of the senescence-associated secretory phenotype (SASP) in lung cells. To elucidate its mechanism of action, the authors synthesized an activity-based probe (probe 22) by attaching an alkyne group to the carboxyl group of CA via an amide bond [83]. Competitive experiments showed that CA effectively reduced the fluorescence signal of proteins labeled by an iodoacetamide probe, confirming that CA forms a covalent bond with cysteine residues in proteins through a Michael addition reaction involving its unsaturated enone structure. Based on these findings, the authors conducted a cysteine reactivity-based proteomic analysis using a desthiobiotin iodoacetamide (DBIA) probe and identified Annexin A5 (ANXA5) as a potential target of CA. To determine the specific binding site of CA on ANXA5, the authors incubated probe 22 separately with wild-type ANXA5 and its C316A mutant (where cysteine 316 was mutated to alanine), followed by fluorescent labeling via click chemistry. The results showed a significant fluorescence signal for wild-type ANXA5 but almost no signal for the mutant, demonstrating that CA covalently binds to Cys316 of ANXA5. Further mechanistic studies revealed that CA binds to Cys316 of ANXA5 and induces its degradation, leading to the inactivation of PKCθ and subsequent inhibition of the NF-κB inflammatory pathway in senescent cells. In another study on CA [84], the same probe (probe 22) was used to identify aconitate decarboxylase 1 as a target of CA. The study revealed that CA exerts neuro-anti-inflammatory effects and alleviates epilepsy symptoms by directly binding to this enzyme and inhibiting the PERK-NF-κB signaling pathway it mediates. Similarly, dihydrocaffeic acid (DA) was also shown to target transaldolase 1 (TALDO1) via an orthogonal probe strategy (probe 23), exerting anti-inflammatory activity by regulating the downstream PERK-IκBα-NF-κB signaling pathway [85].

Rosmarinic acid (RA) is a natural polyphenolic compound with various pharmacological activities, including anti-inflammatory [91], antitumor [92], and neuroprotective effects [93]. Fan’s team found that RA can inhibit doxorubicin (DOX)-induced senescence in cardiomyocytes. To investigate the underlying mechanism, they synthesized probe 24 by linking the carboxyl group of RA to propargylamine via an amide bond [86]. Using this probe in combination with proteomic analysis, the researchers identified the 14-3-3θ protein as a potential target of RA in senescent HL-1 cells. Subsequently, the authors further confirmed the direct interaction between RA and 14-3-3θ through cellular thermal shift assay (CETSA), microscale thermophoresis (MST), in-gel fluorescence analysis, molecular docking, and site-directed mutagenesis experiments. Mechanistic studies revealed that RA upregulates the expression of 14-3-3θ, enhances Foxo1 phosphorylation, inhibits its nuclear translocation, thereby attenuating the activation of downstream senescence-related signaling, and alleviates DOX-induced cardiomyocyte senescence. In another study on myocardial ischemia–reperfusion injury (MI/RI) [87], the researchers also used probe 24 for target screening and found that ubiquitin-specific peptidase 15 (USP15) is a potential target of RA. RA binds to USP15, activating the Keap1/Nrf2 signaling pathway and subsequently mitigating MI/RI.

Chlorogenic acid (CGA) is a natural product commonly found in sources such as green coffee beans. Screening studies have identified its anti-aging activity. To investigate the mechanism of action, the research team utilized a click chemistry-based probe (probe 25) in combination with proteomic analysis and identified Enolase 1 (ENO1) as a key target of CGA in human dermal fibroblast (HDF) cells. Further studies demonstrated that CGA binds to and inhibits the activity of ENO1, thereby blocking the glycolytic pathway and preventing UVA-induced cellular senescence [88]. Furthermore, to elucidate the anticancer mechanism of CGA, Wang’s team designed and synthesized probe 25 [89]. Using proteomic methods, the researchers isolated and identified mitochondrial acetyl-CoA acetyltransferase 1 (ACAT1) as its main target and confirmed that CGA inhibits cancer cell proliferation by suppressing the phosphorylation of tetrameric ACAT1.

**Figure 8:**
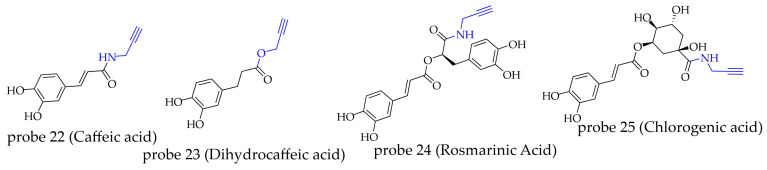
The structures of labeled probes derived from phenylpropanoid-based natural products.

Phenylpropanoid compounds generally possess relatively compact molecular structures. Excessively large tagging groups can interfere with their binding activity, making smaller-volume alkyne groups more suitable for application. Regarding the choice of modification sites, the side-chain C3 position is commonly selected. Unlike the modification at the phenolic positions in flavonoids mentioned earlier, the phenol or catechol structures in phenylpropanoids contribute significantly to their biological activity (Figure 8).

### 4.5. Research on Other Natural Products

In addition to the aforementioned classes of natural products, many naturally occurring compounds exhibit remarkable structural diversity. Some are as simple as short carbon chains bearing active groups such as carboxyl groups, while others contain multiple conjugated benzene rings or even represent composite structures integrating features of the previously mentioned categories. This rich structural diversity not only gives rise to a broader spectrum of biological activities but also poses greater challenges for the targeted modification of natural products (Table 5).

Aristolochic acid (AA) is a nitrophenanthrene carboxylic acid compound derived from plants of the *Aristolochia* genus, possessing strong nephrotoxicity and carcinogenicity [106]. To investigate its toxic mechanisms, one research team synthesized an orthogonal probe (probe 26), which retained toxicity similar to that of AA sodium salt [94]. Using proteomics, they identified multiple key enzymes involved in mitochondrial respiration and metabolism, including IDH2, MDH2, PKM, LDH, FASN, HK2, and ATP synthase, preliminarily revealing potential metabolic disruption mechanisms underlying AA-induced nephrotoxicity. In another study focusing on the nephrotoxicity of AA, Gong’s team synthesized a photo-crosslinking probe (probe 27) [95]. This probe exhibited toxicity comparable to that of natural AA. Proteomic analysis identified 32 high-confidence binding proteins, and further screening showed that only knockdown of HIGD1A, which is localized to mitochondria, exacerbated AA-induced cell death. Subsequent validation using surface plasmon resonance (SPR), isothermal titration calorimetry (ITC), and cellular thermal shift assay combined with Western blot (CETSA-WB) confirmed the direct binding between AA and HIGD1A. Molecular docking further identified Met72 as the key binding site. Mechanistic studies indicated that AA binds to HIGD1A, specifically destabilizing the HIGD1A-TFMA protein complex, leading to TFAM degradation, triggering mitochondrial DNA release, and ultimately activating the MAPK/NF-κB pathway, which induces inflammatory responses and renal tissue damage. The two studies employed different labeling groups for the same natural product and indication yet identified distinct key targets and mechanisms, with each set of results experimentally validated. This reflects the complexity of the mechanisms of action of natural products and also underscores the limitations of current probe technologies that no single probe can fully capture all relevant targets.

Cannabidiol (CBD), a non-psychoactive cannabinoid derived from cannabis, shows potential in treating methamphetamine (METH) addiction, though its precise mechanism of action remains unclear. To address this, a research team designed and synthesized a photoaffinity probe (probe 28) for CBD [96]. This probe utilizes benzophenone as the photo-crosslinking group, with CBD and an alkyne handle attached at its two ends. The researchers first verified the comparable bioactivity of probe 28 and CBD through a conditioned place preference (CPP) test. In the target capture experiment, cells were irradiated with 365 nm UV light to activate the benzophenone group in probe 28, enabling covalent linkage with target proteins. Subsequently, azide-biotin was added to the cell lysate, and a copper-catalyzed azide-alkyne cycloaddition (CuAAC) reaction was employed to introduce the biotin tag into the probe-protein complex. The complex was then enriched using streptavidin-agarose beads. The extracted proteins were separated by SDS-PAGE, visualized by silver staining, identified by mass spectrometry and analyzed via Gene Ontology (GO) analysis based on cellular components, which identified the ATP synthase alpha subunit (ATP5A1) as a key target. Mechanistic studies revealed that CBD binds to ATP5A1, counteracts METH-induced ubiquitination of ATP5A1, promotes the assembly of the ATP synthase complex, and inhibits METH-triggered addictive behavior through the ADO-A1R signaling pathway. Another study focused on cannabinol (CBN) [97], using a minimalist diazirine-based probe (probe 29) to reveal its neuroprotective effect through targeting mitochondrial isocitrate dehydrogenase 2 (IDH2). The two studies on structurally distinct cannabinoids employed similar sites for probe modification, further demonstrating that probe design strategies for compounds of the same class possess reference value and transferability.

Fructose-1,6-bisphosphate (FBP) is an endogenous metabolite involved in the glycolytic pathway. It can also function as a signaling molecule by binding to key proteins and regulating various protein-related events. To systematically map its interaction network, Li and his team designed and synthesized a photoaffinity probe (probe 30) from scratch [98]. The probe was constructed by linking the minimal linker L1 to the C-4 position of FBP via an ester bond. In-gel fluorescence scanning revealed that probe 30 labeled more proteins when incubated with live cells than with cell lysates, suggesting better reactivity in a live-cell context. Therefore, the authors incubated probe 30 with live HepG2 cells, activated photo-crosslinking with 365 nm light for 5 min, and then lysed the cells. Azide-biotin was introduced via click chemistry, and the probe-protein complexes were enriched using streptavidin-coated agarose beads. After trypsin digestion, LC-MS/MS was used for detection and identification. Many previously unreported proteins that interact with FBP were identified, including the mitochondrial metabolic enzyme aldehyde dehydrogenase 2 (ALDH2). FBP was shown to inhibit ALDH2 activity, leading to increased cellular reactive oxygen species (ROS) and mitochondrial fragmentation.

**Figure 9:**
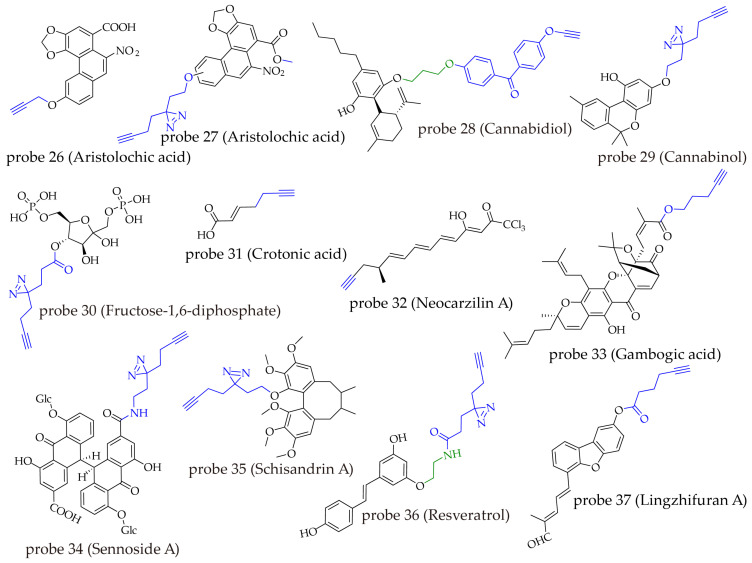
The structures of labeled probes derived from other natural products.

In addition to the aforementioned work, probe studies on other natural products have also frequently achieved success, indicating that the preparation of such probes is not necessarily complicated, even when the parent compounds possess complex structures. The current strategies can be broadly classified into two categories: one involves alkyne substitution for chain-like structures, such as crotonic acid (probe 31) [99] and neocarzilin A (probe 32) [100]; the other employs tag conjugation via readily modifiable chemical groups, as seen with gambogic acid (probe 33) [101], sennoside A (probe 34) [102], schisandrin A (probe 35) [103], resveratrol (probe 36) [104], and lingzhifuran A (probe 37) [105]. This suggests that for natural products with intricate architectures, commencing modifications from chemically accessible sites serves as a key breakthrough to enhance research efficiency (Figure 9).

## 5. Summary and Future Perspectives

Overall, the applicability of the labeling groups discussed above is largely governed by two key factors: their molecular size and binding properties. For natural products with relatively small molecular volumes, strategies such as alkyne modification or the use of minimalist diazirine-based probes are more commonly employed. In contrast, labeling larger natural products often requires careful consideration of steric hindrance introduced by the parent structure, which may interfere with the function of the labeling moiety. In such cases, incorporating a linker of appropriate length becomes essential, and bulkier photoaffinity labels like benzophenone tend to offer better compatibility and performance. Analysis of labeled probe applications across various classes of natural products reveals several empirical patterns. For the same natural compound, different therapeutic indications may necessitate distinct labeling strategies—exemplified by berberine, which has been conjugated with benzophenone, aryl-diazirine, and aryl-azide tags in separate studies. Conversely, the same probe can be successfully applied across diverse disease models; probes derived from celastrol, eupalinolide B, and rosmarinic acid, for instance, have facilitated the discovery of new targets in multiple pathological contexts. These variations likely stem from differences in binding modes between the natural product and its target proteins, as well as the distinct microenvironments of their respective binding pockets. Further analysis indicates that, among structurally related natural products, modification sites can often be inferred from precedents within the same class. For example, flavonoids are typically modifiable at phenolic hydroxyl groups without significant loss of activity, whereas phenylpropanoids are more amenable to modification at the C3 side chain. Such structure–activity relationship trends offer valuable guidance for probe design and mechanistic studies of unexplored analogues.

Despite the proven utility of labeled probes in elucidating the mechanisms of action of natural products, no single labeling group currently enables comprehensive coverage of all potential targets. Moreover, chemical modification for labeling purposes often results in only partial retention of the original bioactivity, and binding affinity may be compromised to varying degrees. Therefore, candidate targets identified through probe-based screening should be further validated using complementary binding assays and functional studies to ensure robustness. This review also highlights recent advances in photoaffinity label development. Based on these emerging trends, we propose that future probe design should prioritize the following aspects: (1) Minimalist design: Reducing the molecular size of labeling groups to minimize structural perturbation of the natural product and improve compatibility with sterically constrained binding sites; (2) Enhanced binding affinity: Optimizing the electronic and structural properties of probes to strengthen interactions with target proteins and improve labeling efficiency; (3) Improved stability: Designing photoaffinity labels with greater chemical and photochemical stability to ensure reliable performance under diverse experimental conditions. These directions aim to maximize labeling efficiency and enhance the reliability of target identification in natural product research.

## Figures and Tables

**Table 1:** Applications and discoveries of probes in alkaloid compounds.

Probe Number	Natural Product	Labeling Group	Biological Activity	Key Findings (Targets)	Reference
1	berberine	benzophenone	anti-inflammatory effect	EIF2AK2	[38]
2	berberine	aryl diazirine	inhibition of phosphorylated JNK.	MAP2K7	[39]
3	berberine	aryl azide	hypoglycemic effect	TIGAR	[40]
4	capsaicin	alkynyl	anti-sepsis effect	PKM2, LDHA and COX-2	[41]
			anti-rheumatoid arthritis effect	PRDX2	[42]
5	tetrandrine	diazirine	antiviral effect	LIMP-2	[43]
6	dopamine	alkynyl	anti-cancer effect	endoplasmic reticulum proteins (ER)	[44]
7	dopamine	alkynyl	anti-parkinsonian effect	aldolase A, α-enolase and pyruvate kinase M2 (PKM2)	[45]
8	hypoxanthine	diazirine	anti-insulin resistance effect	AMPK/mTOR/PPARα pathway	[46]
9	cephalosporin	alkynyl	antibacterial effect	virulence-associated enzyme ClpP and a resistance-associated β-lactamase	[47]
10	tetrodotoxin	aryl diazirine	sodium channel blockade		[48]

**Table 2:** Applications and discoveries of probes in terpenoid compounds.

Probe Number	Natural Product	Labeling Group	Biological Activity	Key Findings (Targets)	Reference
11	ganoderic acid T	biotin	anti-liver cancer effect	phosphoenolpyruvate carboxykinase (PC)	[59]
12	pedunculoside	biotin	anti-pathological cardiac hypertrophy effect	GATA6	[60]
13	celastrol	alkynyl	anti-liver fibrosis effect	peroxiredoxins (PRDXs) and heme oxygenase 1 (HO-1)	[61]
			anti-breast cancer effect	mitochondrial isocitrate dehydrogenases (IDHs)	[62]
			antimalaria effect	PfSpdsyn and PfEGF1-α	[63]
			cardiotoxicity	AMOTL2	[64]
14	eupalinolide B	alkynyl	anti-triple-negative breast cancer effect	branched-chain amino acid transaminase 1 (BCAT1)	[65]
			anti-periodontal inflammatory effect	UBE2D3	[66]
15	glaucocalyxin A	alkynyl	anti-renal cell carcinoma effect	PRDX1/2	[67]
16	asiatic acid	diazirine	anti-liver cancer effect	α-tubulin alpha-1B chain (TUBA1B)	[68]
17	pseudolaric acid A	diazirine	anti-cancer effect	methylenetetrahydrofolate dehydrogenase 1-like (MTHFD1L)	[69]
18	lactucin	alkynyl	anti-lung adenocarcinomata	PGM, PKM, LDHA and PDH	[70]

**Table 3:** Applications and discoveries of probes in flavonoid compounds.

Probe Number	Natural Product	Labeling Group	Biological Activity	Key Findings (Targets)	Reference
19	puerarin	diazirine	anti-obesity effect	GABRA1	[76]
			anti-cardiovascular disease effect	CHAF1B	[77]
20	norathyriol	alkynyl	inhibition of miRNA	AGO2	[78]
21	bavachinin	alkynyl	anti-nonalcoholic fatty liver disease effect	proliferating cell nuclear antigen (PCNA)	[79]

**Table 4:** Applications and discoveries of probes in phenylpropanoids.

Probe Number	Natural Product	Labeling Group	Biological Activity	Key Findings (Targets)	Reference
22	caffeic acid	alkynyl	anti-idiopathic pulmonary fibrosis effect	ANXA5	[83]
			anti-seizures effect	aconitate decarboxylase 1	[84]
23	dihydrocaffeic acid	alkynyl	anti-acute pneumonia effect	transaldolase 1(TALDO1)	[85]
24	rosmarinic acid	alkynyl	anti-DOX-induced cardiotoxicity effect	14-3-3θ	[86]
			anti-myocardial ischemia–reperfusion injury (MI/RI) effect	ubiquitin-specific peptidase 15 (USP15)	[87]
25	chlorogenic acid	alkynyl	anti-photoaging effect	enolase 1 (ENO1)	[88]
			anti-cancer effect	acetyl-CoA acetyltransferase 1 (ACAT1)	[89]

**Table 5:** Applications and discoveries of probes in other natural products.

Probe Number	Natural Product	Labeling Group	Biological Activity	Key Findings (Targets)	Reference
26	aristolochic acid	alkynyl	aristolochic acid nephropathy	IDH2, MDH2, PKM, LDH, FASN, HK2 and ATP synthase	[94]
27	aristolochic acid	diazirine	aristolochic acid nephropathy	HIGD1A	[95]
28	cannabidiol	benzophenone	anti-methamphetamine addiction	ATP synthase alpha subunit (ATP5A1)	[96]
29	cannabinol	diazirine	neuroprotection	isocitrate dehydrogenase 2 (IDH2)	[97]
30	fructose-1,6-bisphosphate	diazirine	mitochondrial fragmentation	ALDH2	[98]
31	crotonic acid	alkynyl	involved in protein post-translational modifications	HDAC	[99]
32	neocarzilin A	alkynyl	antiproliferative and antimigratory	reticulon 4 (Rtn4)	[100]
33	gambogic acid	alkynyl	anti-lung cancer	6PGD	[101]
34	sennoside A	diazirine	laxative effect	NfrA	[102]
35	schisandrin A	diazirine	neuroprotection t	Ykt6	[103]
36	resveratrol	diazirine	inhibition of cervical cancer metastasis	fatty acid binding protein 5F(ABP5)	[104]
37	lingzhifuran A	alkynyl	anti-triple-negative breast cancer	PABPC1	[105]

## Data Availability

No new data were created or analyzed in this study. Data sharing is not applicable to this article.
